# Society Meetings

**Published:** 1916-02

**Authors:** 


### SOCIETY MEETINGS

**Brief reports and announcements of meetings of societies of Western New York, and those of general scope, are requested from Secretaries. Copy should be on hand the fifteenth of the month. Full transactions will be published at cost of composition.**

---

**DOCTOR: Is your Society properly represented here? If not, please bring our standing announcement to the attention of the members.**

---

**Buffalo Academy of Medicine,** Section of Pathology, Dec. 28, X-ray Plates of Some Interesting Heart and Lung Conditions in Children, Dr. Irving M. Snow and Dr. Frank N. Potts; Dr. Baldwin Mann, Report of Blood Cultures Observed in Pathologic Laboratory of Roosevelt Hospital, May, 1909 to Jan. 1915.

Section of Surgery, Jan. 5, Diagnosis of Renal and Ureteral Calculi, with X-ray Plates, Dr. Hugh Cabot, Boston.

Section of Medicine, Jan. 12, Pathologic Diagnosis, Dr. B. T. Simpson; Importance of Dietetic and Medical Means in Prevention of Constitutional Diseases—Scurvy, Spasmophilic Diathesis, Exudative Diathesis and Rickets, Dr. H. J. Gerstenberger, Prof. of Paediatrics, Western Reserve University, Cleveland.

Section of Obstetrics and Gynaecology, Jan. 19, Prolapse of Uterus, Dr. Channing W. Barrett, Chicago. (Lantern slides).

---

**The Medical Society of the County of Niagara** held a banquet Jan. 12 and installed the following officers: Pres., H. A. Wilmet, Middleport; V. P., John C. Plain, Ransomeville; Sec., L. C. Hurlbut, Lockport; Treas., Walter A. Scott, Niagara Falls; to 8th Dist. Branch, Edwin Shoemaker, Newfane.

---

**Rochester Academy of Medicine.** Annual Meeting, with Section I, Jan. 12. Abdominal Diagnosis, Dr. Charles D. Young; Clinical Significance of Abdominal Ptoses, Dr. John M. Swan. Officers were elected as follows: President, Joseph R. Culkin;

Vice-Presidents, Robert T. French, Charles W. Hennington, Lucius L. Button, Myron B. Palmer; Treasurer, Nathan D. McDowell; Trustees, William V. Ewers, Charles A. Darrow, Joseph W. Magill; Councillors, John M. Swan, George W. Goler; Member Library Committee, Charles W. Hennington.

---

### Medical Association of Central New York.

Minutes of 47th Annual Meeting, continued from last issue.

The PRESIDENT: I think in spite of the fact that we have but a few minutes before luncheon we will proceed to the business meeting. The first thing is the reading of the minutes of the previous meeting.

The secretary read the minutes of the last meeting.

Vice-President Benedict in the Chair.

The VICE-PRESIDENT: You have heard the minutes of the last meeting read:. Are there any corrections?

It was moved and seconded that the minutes of the last meeting be approved as read, and filed.

Carried.

President Boswell in the chair.

The Secretary read a communication from the State Medical Journal, addressed to the Secretary, regarding the publication of the Proceedings of the Association.

DR. DOWD, Buffalo: I heard this discussion in Syracuse last year, and of course we all realize there was not one single paper published in the New York State Journal last year. Now, this society is limited to Central and Western New York, west of Syracuse and as far as the Pennsylvania line. In this district we have a medical journal which until a very few years ago, published not only most of the papers, but the Transactions; in fact, there was a book issued each year containing everything. I cannot see why it would not be a good idea to go back to that same plan. Therefore, to bring this before the meeting, I will make a motion that it be the concensus of opinion of the members of the Central New York State Medical Society that they have a representative journal for the publication of their Transactions, also for the publication of their papers, and that the Buffalo Medical Journal be designated as such.

The PRESIDENT: I think, Doctor, we will take that up under the head of New Business. I will now call for the report of the Treasurer, Dr. Laurie.

Treasurer's report read.

It was moved and seconded that the Treasurer's report be accepted. Carried.

The PRESIDENT: Before I forget it, I want, in the name of the Society, to thank the Rochester Medical Association for the use of this building for today's meeting. I think the building has proven to us in Rochester its use as a central gathering point for all the professional men in the city, and I am sure it has made a very convenient and desirable place for this Society to hold its meeting, and therefore in the name of the Society I will thank the Rochester Medical Association for the use of this building. Are the Credential Committe ready to report?

The SECRETARY: There are two names to be acted upon. The two names that are eligible are Dr. Stark and Dr. Leiter, both of Syracuse. I move you, Mr. President, in view of the fact that the Credential Committee is not here, that these men be added to the list of membership of the Society.

Motion seconded and carried.

The PRESIDENT: Has the Publication Committee anything to report?

The SECRETARY: Nothing.

By direction of the President the Stenographer read the motion put by Dr. Dowd in regard to publication of the Proceedings of the Society. (See previous page.)

The PRESIDENT: Is the motion seconded?

Motion seconded.

The PRESIDENT: It is moved and seconded that the Proceedings be published annually, and that the Buffalo Medical Journal be designated to publish such proceedings. Before the discussion opens I should like to call upon some one to give us some idea of the expense that would involve, what they would expect us to do, and some information on the subject.

DR. BENEDICT, Buffalo: Mr. President, I am not prepared to make a definite proposition, but in the past we have made this general offer to societies in the territory of Western New York, that we would publish free the papers just as they would be accepted if contributed privately, and I am very happy to say that such a program as this is practical and available in its entirety, except for the fact that we cannot very well print the beautiful lantern slide demonstrations and some things of that sort, but so far as such material is presented that can be printed it will be. However, for any detail work, which deals with transactions or business reports or matters of that sort, which are not interesting as reading matter to the profession generally, we would expect to receive about what we have got to hand over immediately to the printer, and that of course would be determined on a pro rata basis, and would be the equivalent of about two dollars a printed page, of something like five hundred words. Now, as to obligations, etc.; if re-prints were wanted, as has been the case several years back, that would be simply a transaction with the printer. The Journal does not furnish re-prints and does not make any profits on them, and has no business interest in them whatever. The Journal would expect to benefit simply by extending its influence as a professional medium, and in such indirect ways as are perfectly obvious.

The PRESIDENT: Having heard this statement of Dr. Benedict, I should like to hear from some of the members on the advisability of undertaking this step. We cannot let such an important matter as this go to a vote without something being said about it.

DR. STOCKTON: I move to refer the matter to the Publication Committee for further consideration.

Amendment seconded.

The PRESIDENT: The amendment is moved to the original motion, that this subject be referred to the Publication Committee for their future communication.

DR. STOCKTON: Refer it to them with power.

The PRESIDENT: The amendment is, that the amendment be referred to the Publication Committee with power to act.

Amendment seconded.

The PRESIDENT: The vote will be taken first on the amendment.

Amendment put to a vote and unanimously carried.

The PRESIDENT: We will now vote on the original motion, that the Society annually publish its transactions, and the Buffalo Medical Journal be designated to so publish them. The question as amended is before you for action. What will you do, gentlemen?

Put to a vote, and carried. *

The PRESIDENT: Dr. Percy, you said you wished to say something to the Society?

DR. PERCY: I want to speak about membership in the Rochester Medical Association. I happen to be Chairman of that committee, and we have three classes of membership, active, associate and non-resident. The dues for non-resident membership are ten dollars annually, and it gives the member the privilege of using this club-house, the cafe and library, and having all the privileges of any active or resident member If there are any here who would like to join we would be glad to receive their applications.

The PRESIDENT: We will now proceed to any further new business. Nominations for the office of president for the ensuing year are now in order.

---

*NOTE:—As the editor was elected President of the Association and, therefore, had to appoint the Committee of Publication, he was placed in a rather embarrassing position. In order to be perfectly impartial, the previous year's committee was re-appointed, without knowledge of their attitude on the question. No influence has been brought to bear on them except that they were urged to make a decision as promptly as possible to prevent undue delay of publication if their vote was favorable—as it has been.

DR. DOWD: Mr. President, we are rotating slowly but surely toward the setting sun, and the next meeting is to be held in Buffalo. It is generally customary to have a man from the city in which the meeting is to be held to represent us, and as Dr. Benedict is Vice-President, I offer his name as President for the ensuing year.

DR. STOCKTON seconded the nomination.

There being no other nominations, it was moved and seconded that the Secretary cast one ballot for Dr. A. L. Benedict of Buffalo for President of the Association for the ensuing year. Carried.

The Secretary cast one ballot for Dr. Benedict for President, and the President declared him duly elected President for the ensuing year.

The President called for nominations for First Vice-President.

DR. ——————: I would like to present the name of Dr. Heazlett, of Auburn, for First Vice-President.

Seconded.

There being no other nominations, it was moved and seconded that the Secretary cast one ballot for Dr. Heazlett of Auburn for First Vice-President of the Association for the ensuing year. Carried.

The Secretary cast one ballot for Dr. Heazlett for First Vice-President, and the President declared him duly elected First Vice-President for the ensuing year.

The President called for nominations for Second Vice-President.

DR. ————— placed in nomination Dr. Edward Wynkoop, of Syracuse, for the office of Second Vice-President.

Seconded.

There being no other nominations, it was moved and seconded that the Secretary cast one ballot for Dr. Wynkoop of Syracuse for Second Vice-President of the Association for the ensuing year. Carried.

The Secretary cast one ballot for Dr. Wynkoop for Second Vice-President, and the President declared him duly elected Second Vice-President for the ensuing year.

The President called for nominations for the office of Secretary.

The SECRETARY: The office of Secretary is held for two years, and no nomination is to be made at this meeting.

The President called for nominations for Treasurer.

DR. —————— nominated Dr. T. F. Laurie, of Auburn, to succeed himself as Treasurer for the ensuing year.

Seconded.

There being no other nominations, it was moved and seconded that the Secretary cast one ballot for Dr. T. F. Laurie of Auburn for Treasurer for the ensuing year.

The Secretary cast one ballot for Dr. Laurie for Treasurer, and the President declared him duly elected Treasurer for the ensuing year.

Recess was here taken for luncheon, the afternoon session being called for 2 o'clock.

AFTERNOON SESSION

The PRESIDENT: We will take up the scientific end of the program again, commencing with the Symposium on Cardio-Vascular Disease.

Drs. George W. Ross and Julian Loudon, of Toronto, Canada, presented the following papers:

(1) Modern Methods of Investigation of Cardio-Vascular Diseases.

a. The Polygraph (with lantern slides).

b. Blood Cultures.

c. The Wasserman Reaction.

d. A Method of Auscultation of the Jugular Veins.

e. The Electro Cardiograph (with lantern slides); X-Rays (with lantern slides).

DR. ROSS: With your permission I will vary the program that is set forth a little bit. One reason is that the X-Ray method is being covered by Dr. Palmer of Rochester, and it perhaps would take me too long to go into all the set parts of this paper. In behalf of Dr. Loudon and myself I wish to thank you for your kindness in asking us to take part in your meeting.

The PRESIDENT: I think we will have Dr. Palmer's paper on the Roentgen diagnosis and then we will take up the discussion of Dr. Ross's, Dr. Loudon's and Dr. Palmer's papers together.

DR. M. B. PALMER, of Rochester, presented a paper entitled "Roentgen Ray Diagnosis of the Heart and Lungs, with special reference to new growths (with lantern slides).

The PRESIDENT: I will ask Dr. Alsener of Syracuse to open the discussion on the methods of diagnosis of the heart and lungs.

DR. ALSENER: Mr. President, Ladies and Gentlemen: It is a great pleasure to discuss these papers, although I will admit to somewhat of a surprise. The subject which is taken up by the first two speakers, the gentlemen from Toronto, certainly has interested us greatly, and they have done us a great favor by coming here and presenting this subject in such an able fashion. Their subjects, the diagnosis of the changed conditions of the heart and the circulation, are of very great interest, not only because of the importance to many of us, particularly to doctors, but also because of the fact that most of it is quite new. It happened to be my good fortune not long ago to spend some time with Dr. Sir James Mackenzie, the man who developed the polygraph about which we have heard this afternoon, the man who was really the pioneer in the study of irregularities of the heart; also to see a good deal of the work of Dr. Thomas Lewis in the electro-cardiographic department of the University Hospital, the one who through his development of what Dr. Mackenzie discovered, through his application of the electro-cardiograph, has been able to tell us that there is such a thing as auricular vibration and tell us about auricular flutter. These two men working together have enabled us to recognize the mild, rather mild, symptoms of endo-carditis. The things which Dr. Mackenzie and Dr. Lewis have enabled us to discard are very numerous indeed, and really one is somewhat humiliated to go over this list of things which have been used by all doctors and recommended by the ablest doctors, and they are now known to be absolutely worthless. In earliest times the detection of an irregularity led on the part of the practitioner to more or less reassurance being given the patient, but without the addition of any knowledge at all to the diagnosis of the case. Now we know that premature beats are, practically speaking, not harmful at all, and very common. We know that heart block, even in the beginning stages, even in the stage in which there is nothing but a prolongation of the interval between the contraction of the auricle and the contraction of the ventricle, that even that beginning stage can be easily diagnosed by either the polygraph or the electro-cardiograph, and that also that that is an important thing to recognize, because a person having that defect in the mechanism of the heart is quite likely to develop superior grades of heart block complications, and which are often fatal complications. That thing being recognized early, that is the time to search for the cause, and may result in a cure or at least prevention of any further progress in the case. I remember Dr. Mackenzie saying one day that having recognized that there was something wrong with the heart one should at once raise this question: Is the heart diseased or is it poisoned? Now, every complication, in the beginning a heart block, is due to some form of poison, possibly nicotine, and possibly something absorbed from the intestines, possibly any one of a number of things we have heard about vaguely, and perhaps many things we have not heard about at all, but much of the cardiac irregularity comes primarily from something which is outside of the heart, which comes through the nervous system rather than the heart itself. Another group of cardiac irregularities, the auricular vibration or flutter cases, have been shown so nicely to respond promptly and quite surely to the proper use of digitalis. These two men have also led us to know that it does not matter so much what preparation of digitalis we use. The old discussion as to whether we use this preparation or that, or this maker or that, is now, if it is not ended it might as well be. There was one point in particular which impressed me as being a very valuable one, and which I think is commonly overlooked. That is this, that the pulse rate should be a very important guide in to a person whose circulation is defective. As was said in the paper, that is now commonly the guide in pulmonary tuber-determining the amount of activity which should be permitted culosis. One does not let a tuberculosis patient overdo just because the fever is gone. So it should be in cardiac cases.

There are many other points in the papers I am sure which might be dwelt on to advantage, but not having in mind making a summary of the thing, I have not a list in my mind, but I feel, Mr. President and Gentlemen, that these papers are of immense value, that they discuss things which we should all be very familiar with, which are of great practical value to our patients.

DR. STOCKTON: Mr. President and Gentlemen: I feel that this is a very admirable illustration of the importance of the newer methods of diagnosis, as they have been presented by Dr. Palmer, by Dr. Loudon and by Dr. Ross. These gentlemen who have come to us from Canada are admirable representatives of their most able clinic in Toronto, and I cannot withhold my word of praise for their two papers. I think Dr. Ross in particular should be congratulated on his ability to state briefly several important matters and to state them so well. I wish to emphasize the importance of the picture which Dr. Ross drew of these cases of infective endo-carditis, and to point out once more the value of what he says in regard to the pulse rate in cases in which there is but moderate variation in temperature, and once more to emphasize his seeming regard for the general appearance and condition and behaviour of his patients. The mental and physical restlessness and irritability, the frequent pulse, the slight temperature and the appearance of illness would attract attention to a case of endocarditis that might otherwise escape serious notice until such time as irreparable damage is done. If nothing else comes of these papers but that picture I think a good deal has been accomplished. I won't take the time for further discussion.

(To be concluded in our next issue).

---

**Indicanuria.** In all cases of indicanuria and highly acid urine due to intestinal acidemia, we are very likely to find traces of serum-albumin and hyaline casts. This does not mean at this time a hopeless nephritis, but it does signify that the injury to the kidney is consequential and must be checked. —Williams.

